# Could medial malleolus fracture be combined with deltoid ligament injury?: A rare case report

**DOI:** 10.1097/MD.0000000000037011

**Published:** 2024-01-26

**Authors:** Mingyan Li, Zihao Liu, Guixian Dong

**Affiliations:** a Department of Orthopaedic Trauma, Harrison International Peace Hospital, Hengshui, Hebei, China.

**Keywords:** deltoid ligament, diagnosis, medial malleolus fracture

## Abstract

**Rationale:**

Medial malleolus injuries mainly comprise of fractures and deltoid ligament ruptures. Medial malleolus fractures, as a kind of common ankle fractures, could occur separately or be accompanied by lateral and posterior malleolus fractures. It is generally agreed that medial malleolus fracture and deltoid ligament rupture could not occur simultaneously.

**Patient concerns:**

In our study, we report a case of 36 year-old man diagnosed with trimalleolar fracture accompanying ankle dislocation initially. The patient was admitted to our hospital due to traffic accident.

**Diagnosis:**

The patient was diagnosed with trimalleolar fracture accompanying ankle dislocation initially. We missed the diagnosis of accompanied deltoid ligament due to the arthralgia of medial ankle and the widened medial articular space in X-ray after operation.

**Intervention:**

As we missed the diagnosis of accompanied deltoid ligament, we only selected open reduction and internal fixation for trimalleolar fracture at first. After we realized the existence of deltoid ligament rupture, the patient refuse further diagnosis and treatment in our hospital.

**Outcomes:**

During the rehabilitation exercise, the patient had medial arthralgia in his right ankle. He complained it and refuse further diagnosis and treatment in our hospital.

**Lessons:**

The newfound injury pattern, medial malleolus fracture accompanying deltoid ligament rupture, has not been reported in previous studies. The injury pattern needs further researches to explore the mechanism and it should be taken seriously in clinical practice.

## 1. Introduction

Ankle fractures have becoming increasingly common in recent years, which make up approximately 9% of all fractures.^[1]^ By 2030, the incidence of ankle injuries is expected to triple rice.^[2]^ Several studies have reported that 25% of ankle fractures include medial malleolus injuries.^[3,4]^ Medial malleolus injuries mainly comprise of fractures and deltoid ligament ruptures. Medial malleolus fractures, as a kind of common ankle fractures, could occur separately or be accompanied by lateral and posterior malleolus fractures. Medial malleolus fractures can be treated conservatively or surgically according to the displacement of fracture.^[5,6]^ Deltoid ligament ruptures generally accompanied by lateral malleolus fractures are divided into superficial ruptures and deep ruptures.^[7,8]^ A deep deltoid ligament injury is equal to a medial malleolus fracture, when complicated with lateral malleolus fracture.^[9,10]^ Either medial malleolus fracture or deep deltoid ligament rupture could lead to widen the medial articular space and thus seriously affect the stability of ankle, which usually needs surgical fixation to avoid posttraumatic osteoarthritis.^[11,12]^

It is generally agreed that medial malleolus fracture and deltoid ligament rupture could not occur simultaneously.^[13–15]^ It is sometimes possible that an anteromedial malleolus avulsion fracture could be accompanied by a deltoid ligament injury.^[16]^ This study reported a failed case of a 36 year-old man diagnosed with trimalleolar fracture accompanying ankle dislocation initially. After surgical treatment, the patient still felt painful in his medial ankle, during the postoperative rehabilitation process. X-ray at postoperative follow-up showed that the medial articular space was widened. And physical examination revealed local tenderness around the medial malleolus. We considered that deltoid ligament rupture may be accompanied at the moment of fracture occurrence and suggested the patient to perform an magnetic resonance imaging (MRI) examination to confirm the diagnosis. But the patient refuse further diagnosis and treatment in our hospital. The aim of this study is to introduce a new injury pattern, medial malleolus fracture accompanying deltoid ligament rupture, which is not recognized by most experts previously.

## 2. Case report

**Figure 1.**
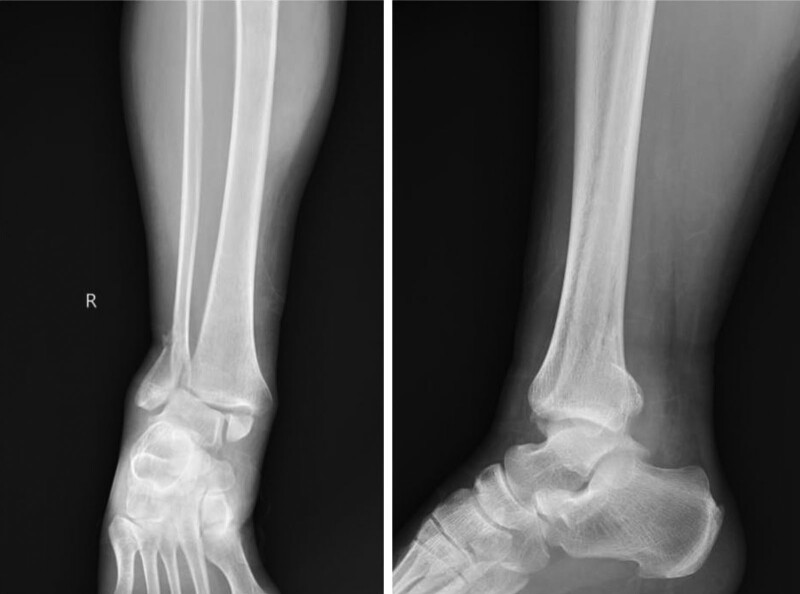
X-ray imaging after injury. It displayed trimalleolar fracture accompanying ankle dislocation.

**Figure 2.**
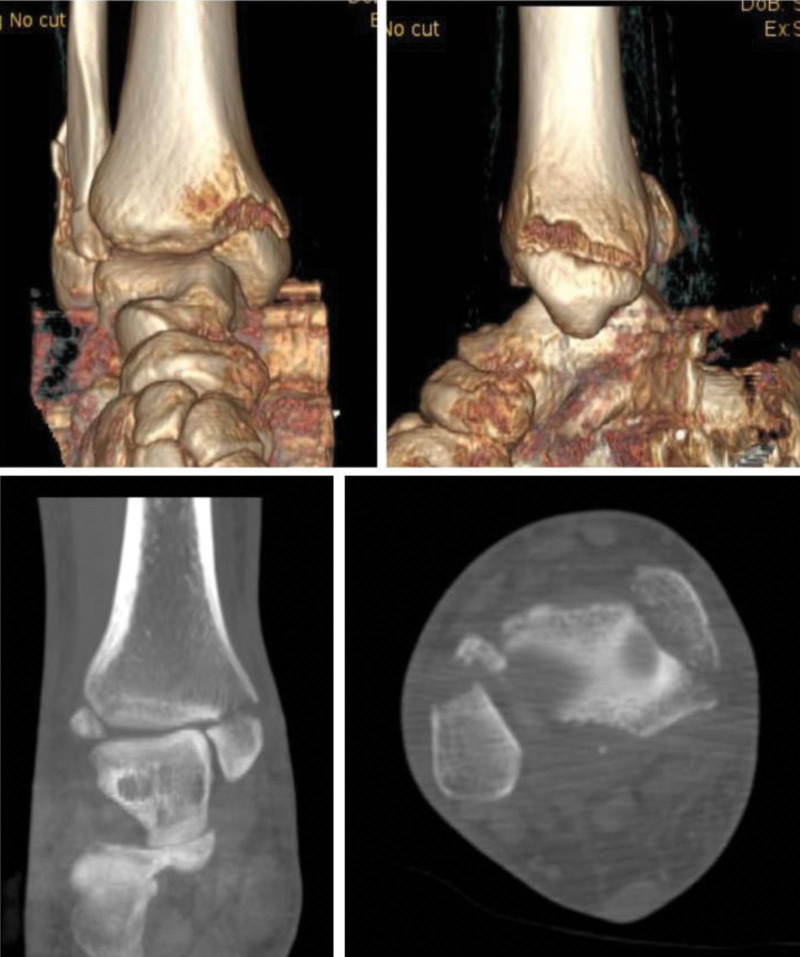
CT imaging after admission. It displayed that the fracture fragment of medial malleolus was large.

In June 2022, a 36-year-old male patient was admitted to the Department of Orthopaedic Trauma, Harrison International Peace Hospital, Hengshui, Hebei Province, China due to traffic accident. Physical examination revealed deformity of right ankle, significant swelling, extensive tenderness, and limited range of motion of his right ankle. He was diagnosed as trimalleolar fracture accompanying ankle dislocation according to X-ray (Fig. 1). After admission, we firstly underwent manipulative reduction and traction support. The deformity of right ankle was corrected. Then we performed further CT examination to define a clear fracture type for designing the operation plan. As the fracture fragment of medial malleolus was large in CT image, we did not take the injury of deltoid ligament into account (Fig. 2). We finally decided to select open reduction and internal fixation for trimalleolar fracture after the swelling was reduced.

**Figure 3.**
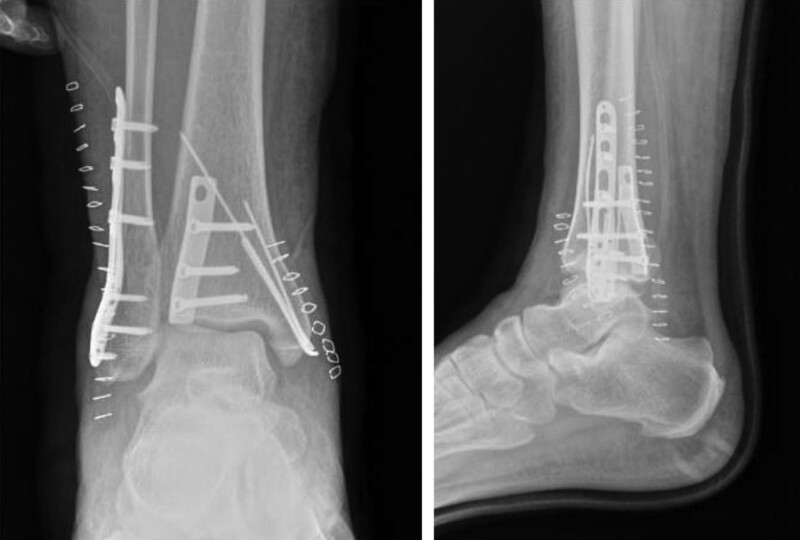
X-ray imaging after surgery. No obvious abnormalities was found.

The patient was required a supine position under spinal anesthesia. Firstly, a posterolateral incision was made to expose lateral and posterior malleolus fractures. After the reduction of fracture, internal fixation by plates and screws was performed for lateral and posterior malleolus fractures. Then a minimally invasive incision was made in medial ankle. After the percutaneous reduction of fracture, one cannulated screw and 2 k-wires fixation was performed for medial malleolus fracture. We did not expose deltoid ligament and perform abduction stress test after fracture fixation during the operation. The final fracture reduction and position of the internal fixation were verified by C-arm fluoroscopy. No obvious abnormalities was found in the joint space (Fig. 3). The right ankle was plastered for maintaining a functional position immediately after surgery.

**Figure 4.**
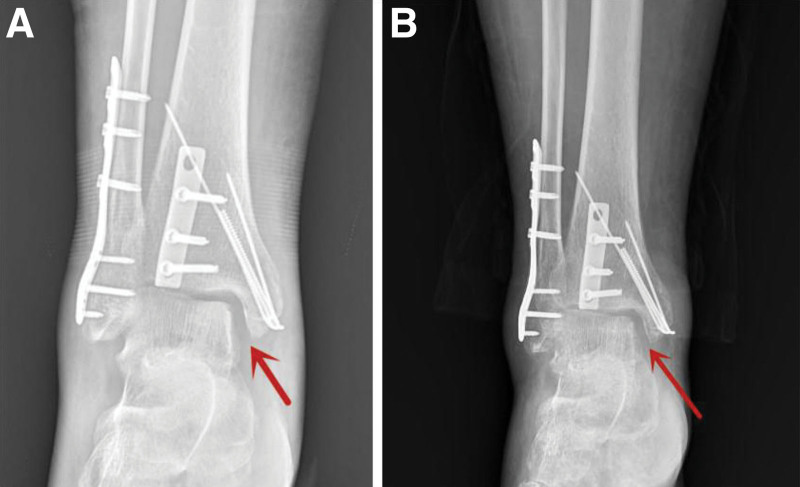
X-ray imaging in his periodic review. (A) Two months after surgery; (B) five months after surgery. The red arrows are pointing to the widen medial articular space.

Four weeks after the surgery, the patient started performing partial weight bearing with the support of plaster. Six weeks after the surgery, the plaster was removed for further rehabilitation exercise. During the rehabilitation exercise, the patient had medial arthralgia in his right ankle. He complained it in his periodic review. Physical examination revealed local tenderness around the medial malleolus. The reexamination X-ray point out the medial articular space was widened (Fig. 4). As no ligament destruction during surgery and no strenuous activity during postoperative rehabilitation, so we considered the patient had got deltoid ligament injury and suggested MRI examination to verify the injury. But the patient refuse further diagnosis and treatment in our hospital. We failed to continue postoperative follow-up of patients.

This study obtained ethical approval from the institutional review board of Harrison International Peace Hospital, Hengshui, Hebei Province, China. The patient provided written informed consent after being fully informed about the treatment and before treatment commenced. The reporting of this study conforms to CARE guidelines.^[17]^

## 3. Discussion

The injury pattern, medial malleolus fracture accompanying deltoid ligament rupture, has not been reported by previous literature.^[18]^ Most orthopedists would overlook the examination of deltoid ligament rupture when encountering patients diagnosed with medial malleolus fracture by interpreting plain radiography. It is generally agreed that the valgus and rotational force could not tear deltoid ligament when medial malleolus fracture has happened.^[19]^ In our study, we report a case of 36 year-old man diagnosed with trimalleolar fracture accompanying ankle dislocation. We failed to realize whether there is a concomitant deltoid ligament injury and it ultimately caused posttraumatic osteoarthritis. Despite the lack of further MRI examination, the arthralgia of medial ankle and the widened medial articular space in X-ray could assist us to indicate the existence of deltoid ligament rupture.

Many studies had proposed the diagnostic criteria of deltoid ligament injury without MRI.^[20–22]^ Pain, swelling, and widen articular space at medial side of ankle joint may highly regarded as having deltoid ligament injury. The definition of widen medial clear space is more than 4 mm on a nonstressed mortise view and at least 1 mm greater than the superior tibiotalar clear space. Our patient complained medial arthralgia during postoperative follow-up. We also found the existence of typical tenderness at medial ankle. His medial clear space displayed widen in postoperative X-ray. As no ligament destruction during surgery and no strenuous activity during postoperative rehabilitation, so we had sufficient reason to believe that the patient had got deltoid ligament injury. Although the injury pattern is seldom seen in clinical practice, we should take it seriously. As for the diagnosis, we should suggest MRI whether the patient accompany medial malleolus fracture. To relieve the financial burden of the patients, we could also draw support from C-arm fluoroscopy to perform abduction stress test after fracture fixation during the operation. If the angle of the talus valgus is >5° on the opposite side, it is considered abnormal. If it is >10°, it can be considered as deltoid ligament injury and repair it immediately. Under non anesthesia, it is often unable to perform due to pain. As for treatment, we suggest surgical repair for complete rupture of deltoid ligament to restore the stability of ankle. The best timing of surgical treatment is primary repair of deltoid ligament to avoid posttraumatic osteoarthritis which has a great influence on restoration of ankle function. As for postoperative rehabilitation, ankle brace or plaster is needed for maintaining a functional position immediately after surgery. The patient could perform partial weight bearing with the support of wearing brace 4 weeks later. Six weeks after the surgery, the brace or plaster could be removed for further rehabilitation exercise.

Deltoid ligament is located on medial ankle, which starts from the lower edge of medial ankle and ends in a fan-shaped structure downwards at the scaphoid, talus, and calcaneus. Deltoid ligament consists of 2 layers: the superficial layer and the deep layer. The superficial layer is the tibia scaphoid ligament, the tibia calcaneal ligament, and the tibia talus superficial ligament. The deep layer is the deep tibial talus ligament, which is closely connected with the Joint capsule. In the currently published study, it is generally admitted that as the deltoid ligament is very strong and tight junction with the Joint capsule, when the medial structure of the ankle joint is strained, the medial malleolus avulsion fracture often occurs. When the avulsion fracture occurs, the deltoid ligament may remain intact. When the fracture fragment of medial ankle is large (fracture line length > 2.8 cm), the deltoid ligament is usually intact. When the fracture fragment of medial ankle is small (fracture line length < 1.7 cm), the deltoid ligament may be teared. Once medial malleolus fracture has happened, deltoid ligament lose one site of attachment point. Hence the valgus and rotational force could not tear deltoid ligament. The case with medial malleolus fracture accompanying deltoid ligament rupture in our study is very rare so that there is no biomechanical studies on this injury pattern. We hope that more and more teams could carry on further researches on this injury pattern to explore the mechanism due to our report. It could help orthopedists to avoid missed diagnosis and choose the appropriate treatment strategies in clinical practice.

There were some limitations in our study. Firstly, we lack MRI examination before and after operative. So we could not confirm the damaged layer which is of significance to explore the injury mechanism. Secondly, we could not entirely exclude the possibility that the deltoid ligament teared during postoperative rehabilitation, although the patient ensured no strenuous activity after surgery.

## 4. Conclusion

In this study we found a new injury pattern, medial malleolus fracture accompanying deltoid ligament rupture, which is not recognized by most experts previously. The missed diagnosis of deltoid ligament rupture caused poor prognosis and dissatisfaction of the patient. This case could help orthopedists to enhance attention to deltoid ligament rupture when encountering patient with medial malleolus fracture.

## Author contributions

**Conceptualization:** Guixian Dong.

**Data curation:** Mingyan Li, Zihao Liu.

**Formal analysis:** Guixian Dong, Zihao Liu.

**Funding acquisition:** Mingyan Li.

**Writing – original draft:** Mingyan Li.
